# Cannabinoid 2 receptor antagonism reverses central nervous system injury-induced immune deficiency syndrome

**DOI:** 10.1186/cc14535

**Published:** 2015-03-16

**Authors:** IB Burkovskiy, J Zhou, G Robertson, C Lehmann

**Affiliations:** 1 Dalhousie University, Halifax, NS, Canada

## Introduction

Central nervous system (CNS) injury, such as stroke, is known to increase susceptibility to infections that adversely affect clinical outcome. This impaired immune response to infection is termed CNS injury-induced immune deficiency syndrome (CIDS). Activation of the cannabinoid 2 receptor (CB2R) suppresses immune function suggesting that antagonism of this receptor may overcome CIDS. The main purpose of this study was to determine the impact of CB2R inhibition on leukocyte activation within the microcirculation following endotoxin challenge in an experimental stroke model.

## Methods

This was a prospective, randomized animal study. Five experimental groups (male C57BL/6 mice, age: 6 to 8 weeks) were subjected to the following treatments: control; endotoxemia (LPS 5 mg/kg, i.v.); transient cerebral hypoxia-ischemia (HI) + endotoxemia; HI + endotoxemia + CB2R antagonist (AM630 2.5 mg/kg, i.v.). HI was induced by unilateral carotid artery occlusion, followed by 50-minute exposure to a low oxygen atmosphere (8% O_2_). The CB2R antagonist was given 15 minutes prior to LPS administration. Intravital microscopy was carried out 2 hours after LPS administration. Brains were then extracted and stained with tetrazolium chloride to calculate the infarct volume. The primary outcome measurement in this study regarding the immune response after stroke was the quantification of leukocyte adhesion following endotoxin challenge in submucosal venules of the gut - an important organ in the development of multiorgan failure in endotoxemia and sepsis.

## Results

Compared with endotoxemic animals without CNS injury, mice subjected to HI displayed reduced leukocyte activation in intestinal submucosal venules indicative of CIDS. Administration of the CB2R antagonist in animals with CIDS challenged with endotoxin restored peripheral leukocyte recruitment without a detrimental impact on infarct size.

## Conclusion

CB2R-related modulation of leukocyte activation is involved in the impaired immune response following CNS injury. Future studies will explore the CB2R pathway in order to develop novel therapies to improve the immune response in CIDS.

